# Usefulness of bioelectrical impedance analysis in multiple sclerosis patients—the interrelationship to the body mass index

**DOI:** 10.3389/fneur.2024.1409038

**Published:** 2024-07-03

**Authors:** Edyta Matusik

**Affiliations:** Department of Rehabilitation, Faculty of Health Sciences in Katowice, Medical University of Silesia, Katowice, Poland

**Keywords:** multiple sclerosis, bioelectrical impedance analysis (BIA), body mass index (BMI), obesity, waist-to-height ratio (WHtR)

## Abstract

**Background:**

Patients with multiple sclerosis (MS) have many potential factors (disease duration, spasticity, immobilization, or glucocorticoid use) that can deteriorate their nutritional status and impact both the progression and prognosis of the disease. Body mass index (BMI), the most widely used nutritional status assessment tool, has important limitations because it does not provide any data on body composition.

**Aim:**

This study aimed to assess the interrelationship between nutritional status assessment by both body mass index (BMI) and body composition using bioelectrical impedance analysis (BIA) and the consistency of diagnosis for underweight/underfat, normal weight/healthy, overweight/overfat, and obesity/obese MS patients.

**Methods:**

Anthropometric [BMI and waist-to-height ratio (WHtR)] and body composition (BIA) data were evaluated in 176 patients with MS. Patients were categorized into four nutritional status subgroups (underweight, normal weight, overweight, obese according to BMI, and underfat, healthy, overfat, and obese according to fat mass% by BIA). The median Expanded Disability Status Scale score was 4.5. Patients were then divided according to EDSS score as mild (EDSS 1.0–4.0) or moderate (EDSS 4.5–6.5) disability subgroups.

**Results:**

Based on BIA assessment, there was a significantly higher prevalence of overfat than of overweight based on BMI [*n* = 50 (28.41%) vs. *n* = 38 (21.59%); *p* < 0.05]. However, the prevalence of obesity did not differ significantly regardless of the mode of diagnosis and was not significantly lower when assessed using BIA [*n* = 26 (14.77%) vs. *n* = 30 (17.05%), respectively]. The overall compatibility rates (CR) of diagnoses made using both BMI and BIA were 75.6, 77.0, and 70.1% for all patients with MS and the mild and moderate subgroups, respectively. The lowest CR was observed in the overweight group. Adiposity significantly underestimated BMI in all subgroups. In the moderate MS subgroup, BMI significantly overcategorized patients with MS as having a normal weight (*p* < 0.05). Stratification for abdominal obesity (WHtR > 0.5) showed that BMI significantly underestimated the prevalence of MS in overweight and obese vs. overfat and obese patients, as assessed using BIA (60.5 vs. 67%; *p* < 0.05). Clinical status (EDSS and ΔEDSS) was more closely related to the nutritional status categorized by FAT% assessed using BIA than using BMI cutoff points. However, the relationship was not statistically significant.

**Conclusion:**

Using the BMI cutoff point for nutritional status assessment in patients with MS is associated with a significant underestimation of excess fat mass. BIA-based FAT% based on BIA have a better relationship with abdominal obesity and disability status than with BMI in patients with MS. The highest rate of false-negative diagnoses was based on the BMI in patients with MS and moderate disability. Adiposity assessment using BIA appears to be a useful method for proper nutritional status assessment in the patients group.

## 1 Introduction

Multiple sclerosis (MS) is caused by an autoimmune process that leads to diffuse demyelination of the central nervous system (CNS) (1). The symptomatology of MS consists of a variety of signs and symptoms, such as weakness and fatigue, spasticity, reduced mobility and ambulation, impaired coordination, sexual dysfunction, and depression. The most commonly proposed theory is that the etiology of MS is related to complex interactions between genetic predispositions and environmental factors. Some of these factors can be modified, which can influence not only the development of the disease but also the progression of disability and prognosis of the treatment outcomes. One of the recently underlined modifiable factors is impaired nutritional status (especially excessive overweight and obesity) (2–5). A study conducted by Hedström et al. in a large-scale Swedish population showed that subjects whose BMI exceeded 27 kg/m^2^ at age 20 had a 2-fold increased risk of developing MS compared to normal-weight persons (4). Moreover, higher depression levels, lower functional capacity, and worse self-rated health status in overweight MS patients were shown by Cambil-Martin et al. (6) compared to the normal-weight MS control group. In a recently published review showing data available over the last 10 years (5), the authors found a significant relationship between obesity onset in pediatric patients and MS development. Body mass index (BMI), the most widely used nutritional status assessment tool, has important limitations because it does not provide any data on body composition. The limitation of using BMI in patients with MS was described by Pilutti et al. (7) and in a review by Dionyssiotis (8), who reported that BMI assessment may underestimate adiposity in patients with multiple sclerosis. However, it is important to distinguish between body weight and fat mass accumulation because of the confirmed relationship between the hormonal function of adipose tissue in obesity (pro-inflammatory adipokine production) and neuroimmunity in patients with MS (9, 10). The two most widely used methods for proper body composition analysis [fat mass (FM), fat-free mass (FFM), and muscle mass (MM)] must be performed: dual energy X-ray absorptiometry (DXA) and bioelectrical impedance analysis (BIA). However, body composition in individuals with MS has not been extensively studied. Moreover, patients with multiple sclerosis (MS) have many other potential factors (disease duration, spasticity, immobilization, or glucocorticoid use) that can deteriorate their anthropometrical status and body composition and may potentially impact both the progression and prognosis of the disease. Our recently published papers confirmed a significant correlation between anthropometric parameters [waist-to-height ratio (WHtR), fat mass, and fat-free mass] and disability level (EDSS) in patients with MS, but body mass index (BMI) was not related to EDSS (11, 12). Despite the increasing availability of bioelectrical impedance analysis (BIA), data showing the usefulness of this method in clinical settings are limited. Therefore, this study aimed to assess the interrelationship between nutritional status assessment using both body mass index (BMI) and bioelectrical impedance analysis (BIA) and the consistency of diagnosis for underweight, normal weight, overweight, and obesity in MS patients.

## 2 Materials and methods

### 2.1 Studied population

In total, 195 patients (132 females/63 males) that were consecutively admitted to the Multiple Sclerosis Management Center were recruited for the study. Subjects who had not experienced an exacerbation within the 30 past days and had no medical conditions, such as cardiac diseases, endocrine disorders, musculoskeletal system diseases, current glucocorticoid therapy, or respiratory diseases, were included. Patients who met the inclusion criteria were included in the final study group (*N* = 176, 128 females/48 males). All the patients had a definite diagnosis of relapsing–remitting or secondary progressive MS according to the McDonald criteria (13) and preservation of at least some ambulatory function [Expanded Disability Status Scale (EDSS) 1.0–6.5, median score 4.5; age 45.68 ± 12.01 years]. The initial EDSS score was obtained retrospectively from medical history at the time of MS diagnosis. The assessment of neurological status on the EDSS was performed by two experienced neurostatus-certified neurologists (EM and BK). Patients were then divided according to EDSS score as mild (EDSS 1.0–4.0) or moderate (EDSS 4.5–6.5) disability subgroup. The baseline clinical and anthropometrical characteristics of the study group is presented in Table 1.

**Table 1:** Baseline clinical characteristics and anthropometrical parameters.

	Stadied population *N* = 176 (F/M = 128/48)			
	Mean	Minimum	Maximum	SD
Age (years)	45.68	20	73	12.01
EDSS initial	2.2	1.0	4.5	0.7
EDSS	3.3	1.0	6.5	1.6
Height (cm)	167.2	152.1	196.1	8.7
Weight (kg)	69.5	40.6	114	16.0
BMI (kg/m^2^)	24.87	16.10	40.30	4.94
Waist c. (cm)	90.4	61.0	126.0	13.5
WHtR	0.54	0.38	0.75	0.08
FM (kg)	20.7	2.6	52.7	9.2
FM (%)	28.8	6.2	48.1	8.4
FFM (kg)	48.9	34.8	75.6	10.1
FFM (%)	71.2	51.9	93.8	8.3

EDSS, Expanded disability status scale; BMI, Body mass index; WHtR, Waist-to-height-ratio; FM, Fat mass; and FFM, Fat-free mass.

### 2.2 Anthropometric measurements and body composition analysis

Anthropometric measurements were recorded on the day of the visit. Standing height was measured to the nearest 0.1 cm. Weight (in underwear) was measured using an electronic scale with readings accurate to 0.1 kg. Body mass index (BMI) was then calculated, using the standard formula (kilograms per meter squared) and classify using standard cut-off points: underweight, BMI < 18.5 kg/m^2^; normal weight, BMI = 18.5–24.9 kg/m^2^; overweight, BMI = 25.0–29.9 kg/m^2^; obese, BMI ≥ 30.0 kg/m^2^. Waist circumference was also measured, and the waist-to-height ratio (WHtR) was calculated. Body composition parameters: fat mass (FM) and fat-free mass (FFM) were assessed [in kilograms (kg) or as percentages of body weight (%)] based on bioelectrical impedance using a leg-to-leg body composition analyzer (BC-420MA Tanita Europe BV, Hoofddorp, The Netherlands) (14). Based on FM%, the patients were classified as underfat, healthy, overfat, or obese. The cut-off points for age and sex are presented in Table 2. All anthropometric and body composition parameters were measured at the same time points as in the subsequent EDSS assessment.

**Table 2:** Fat mass (FM) (%) ranges for adults.

Age (years)	Women			
	Underfat	Healthy	Overfat	Obese
20–39	≤ 21.0%	21.1–33.0%	33.1–39.5%	≥39.6%
40–59	≤ 23.0%	23.2–34.0%	34.3–40.0%	≥40.1%
60–79	≤ 24.0%	24.1–35.2%	35.3–41.5%	≥41.6%
Age (years)	Men			
	Underfat	Healthy	Overfat	Obese
20–39	≤ 7.0%	7.1–20.0%	20.1–25.0%	≥25.1%
40–59	≤ 10.5%	10.6–22.0%	22.1–28.2%	≥28.3%
60–79	≤ 12.2%	12.3–25.0%	25.1–30.0%	≥30.1%

(Based on https://tanita.eu/understanding-YO-measurements/body-fat-percentage).

### 2.3 Ethical considerations

This study was approved by the Ethics Committee of the Medical University of Silesia (approval no. KNW/0022/KB/179/17). All the participants provided informed consent. The patient rights were approved according to the Declaration of Helsinki.

### 2.4 Statistical analysis

Differences in the distribution of each nutritional status category based on BMI and BIA were assessed using the chi-square test. The interrelationship and compatibility rate between the diagnoses made using BMI and BIA in the entire group and disability status subgroups were assessed using frequency tables. Clinical status differences (expressed as EDSS and ΔEDSS) within different nutritional status categories for both BMI and BIA were assessed using one-way ANOVA. All statistical analyses were conducted using the Statistica™ 12 PL software and a *p* value less than 0.05 was considered significant.

## 3 Results

### 3.1 Prevalence of every nutritional status category based on BMI vs. BIA assessment in the entire group of MS patients

**Figure 1:**
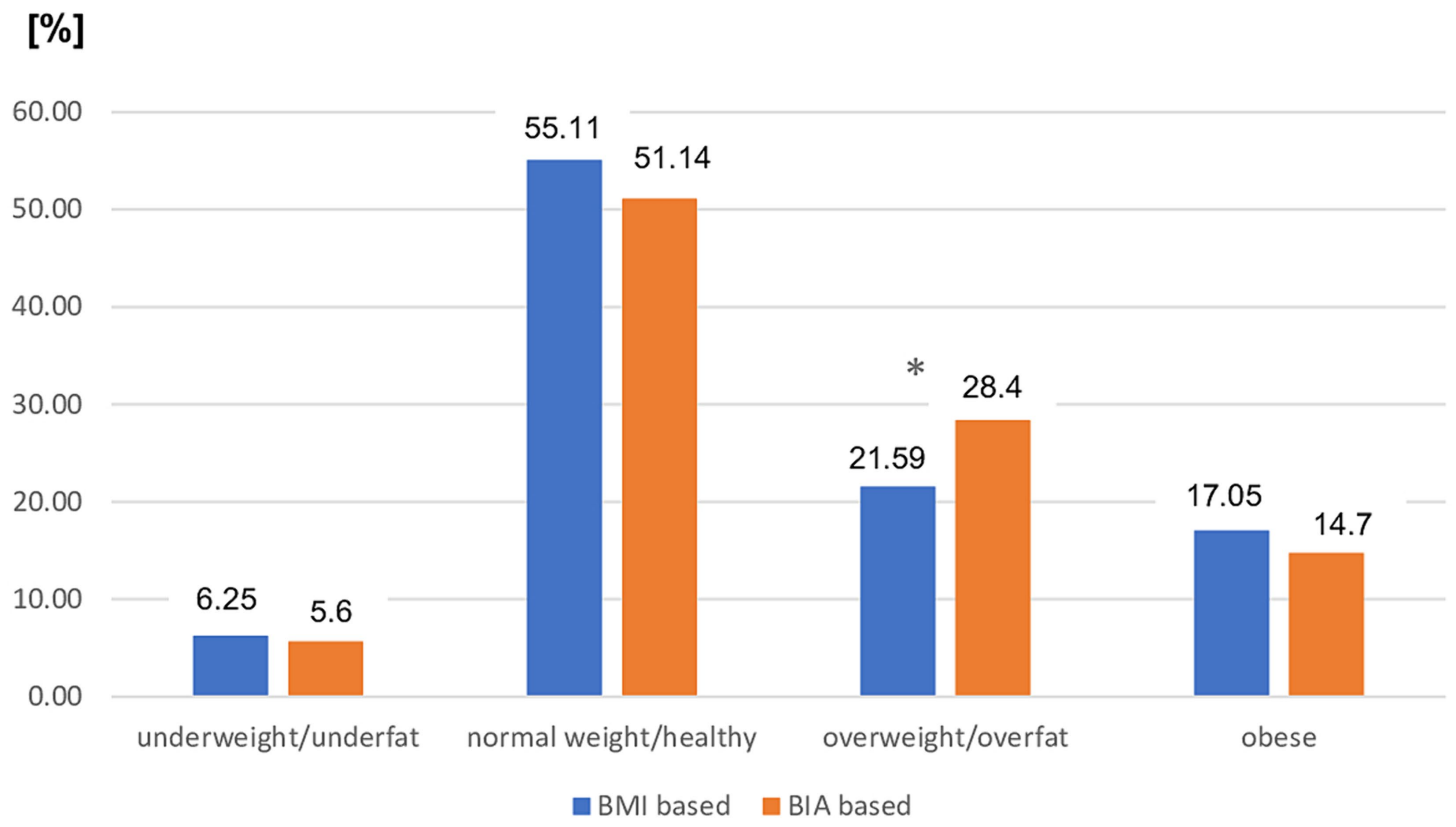
Prevalence of different nutritional status in the entire MS patients group based on BMI vs. BIA. BMI, Body mass index; BIA, Bioelectrical impedance analysis; ^*^p < 0.05 (chi-square test).

The distribution of nutritional status categories diagnosed by BMI cutoff points was as follows: underweight, *n* = 11 (6.25%); normal weight, *n* = 97 (55.11%); overweight, *n* = 38 (21.59%); and obese, *n* = 30 (17.05%). Bioimpedance (BIA) revealed a significantly lower prevalence of underfat and healthy individuals [*n* = 10 (5.69) and *n* = 90 (51.14%), respectively] in the study group. Based on BIA assessment, there was a significantly higher prevalence of overfat than of overweight based on BMI [*n* = 50 (28.41%) vs. *n* = 38 (21.59%); *p* < 0.05]. However, the prevalence of obesity did not differ significantly regardless of the mode of diagnosis and was not significantly lower when assessed using BIA [*n* = 26 (14.77%) vs. *n* = 30 (17.05%), respectively]. The distribution of each nutritional status category for both BMI and BIA assessments is shown in Figure 1.

### 3.2 Compatibility of the nutritional status diagnoses based on BMI vs. BIA assessment in the entire group of MS patients

The overall compatibility rate (CR) of diagnoses made using both BMI and BIA was 75.6% (*n* = 133) for the entire patient group. The lowest CR was observed in the overweight group. Only 71.1% (*n* = 27) of the MS patients diagnosed as overweight by BMI were also overweight by BIA, accounting for only 54% of all overweight MS patients. The best compatibility was found for the healthy category by BIA, in which the CR with a BMI normal weight diagnosis was 90%. Detailed diagnoses based on BMI, BIA interrelationship, and compatibility are presented in Tables 3, 4.

**Table 3:** Interrelationship between different nutritional status diagnosis based on BMI vs. BIA in the entire study group.

Diagnosis by BMI (*n*)	Diagnosis by BIA (*n*)				*BMI all*
	underfat	healthy	overfat	obese	
Underweight	7	4	0	0	*11*
Normal	3	**81**	11	2	***97***
Overweight	0	5	**27**	6	***38***
Obesity	0	0	12	**18**	***30***
*BIA all*	***10***	***90***	***50***	***26***	**133/176 (75.6%)**

BMI, Body mass index; BIA, Bioelectrical impedance analysis. Bold numbers indicate the concordance of diagnoses between the described methods used to identify adequate nutritional status.

**Table 4:** Two-sided compatibility rate for all MS patients.

Nutritional status	All (*n* = 176)		*p*
	BMI vs. BIA	BIA vs. BMI	
Underweight/underfat	63.6% (7/11)	70.0% (7/10)	NS
Normal/healthy	83.5% (81/97)	90.0% (81/90)	NS
Overweight/overfat	**71.1% (27/38)**	**54.0% (27/50)**	***p* < 0.05**
Obesity/obese	60% (18/30)	69.2% (18/26)	NS

BMI, Body mass index; BAI, Bioimpedance analysis. *p* values were determined using the chi-square test. Bold numbers refer to significant differences in the diagnosis of nutritional status between the methods used to assess it.

### 3.3 Prevalence of every nutritional status category based on BMI vs. BIA assessment in mild and moderate disability subgroups of MS patients

**Figure 2:**
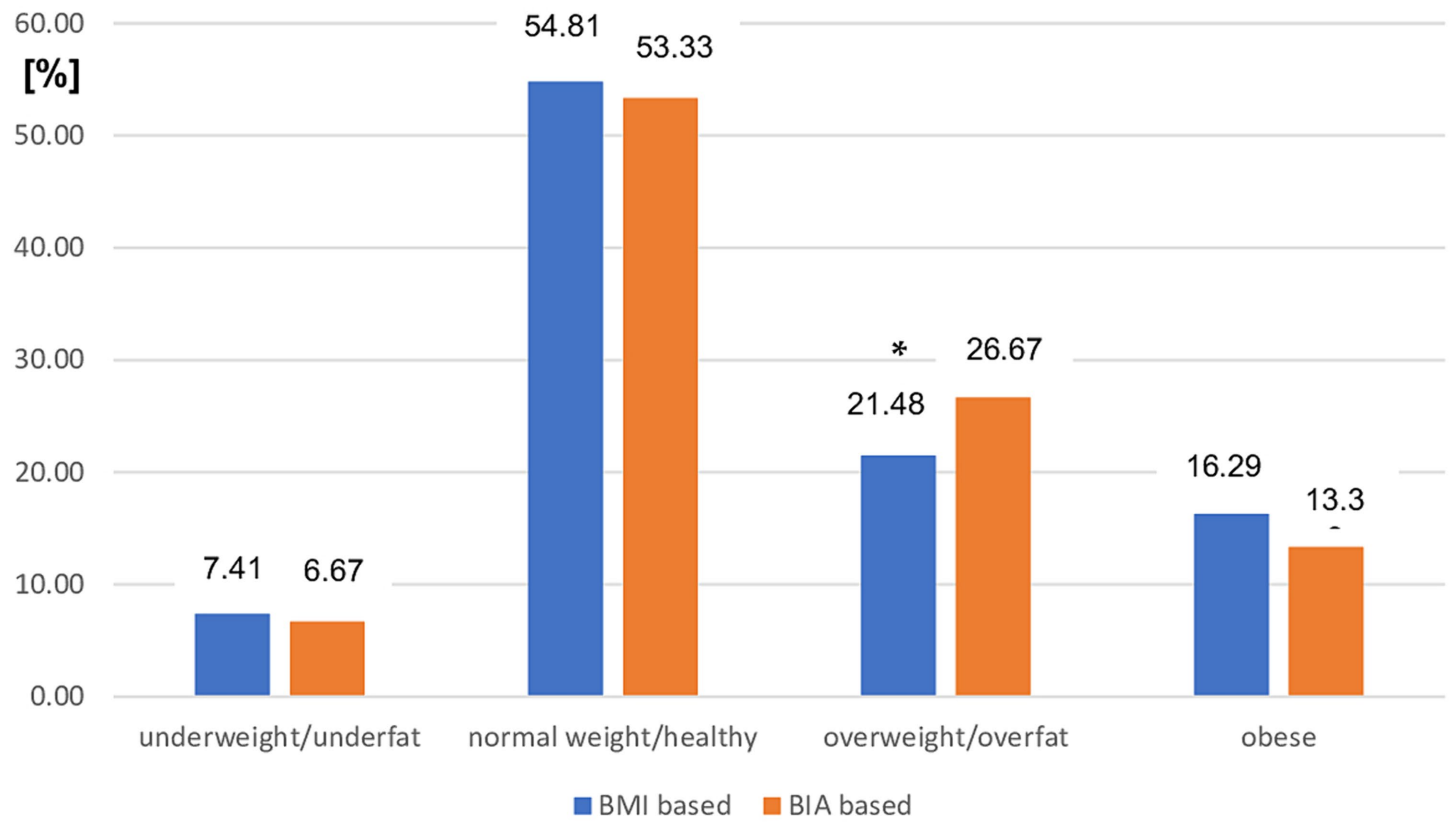
Prevalence of different nutritional status in mild MS patients subgroup based on BMI vs. BIA. BMI, Body mass index; BIA, Bioelectrical impedance analysis; ^*^p < 0.05 (chi-square test).

The distribution of nutritional status categories diagnosed by both BMI and BIA cutoff points in patients with MS with mild disability was similar to that in the entire study group. There was a significant difference between the prevalence of overweight (assessed using BMI) and the overfat category assessed using BIA (*p* < 0.05). The prevalence of other nutritional statuses did not differ significantly between the two modes of diagnosis in this subgroup of patients with MS (Figure 2).

**Figure 3:**
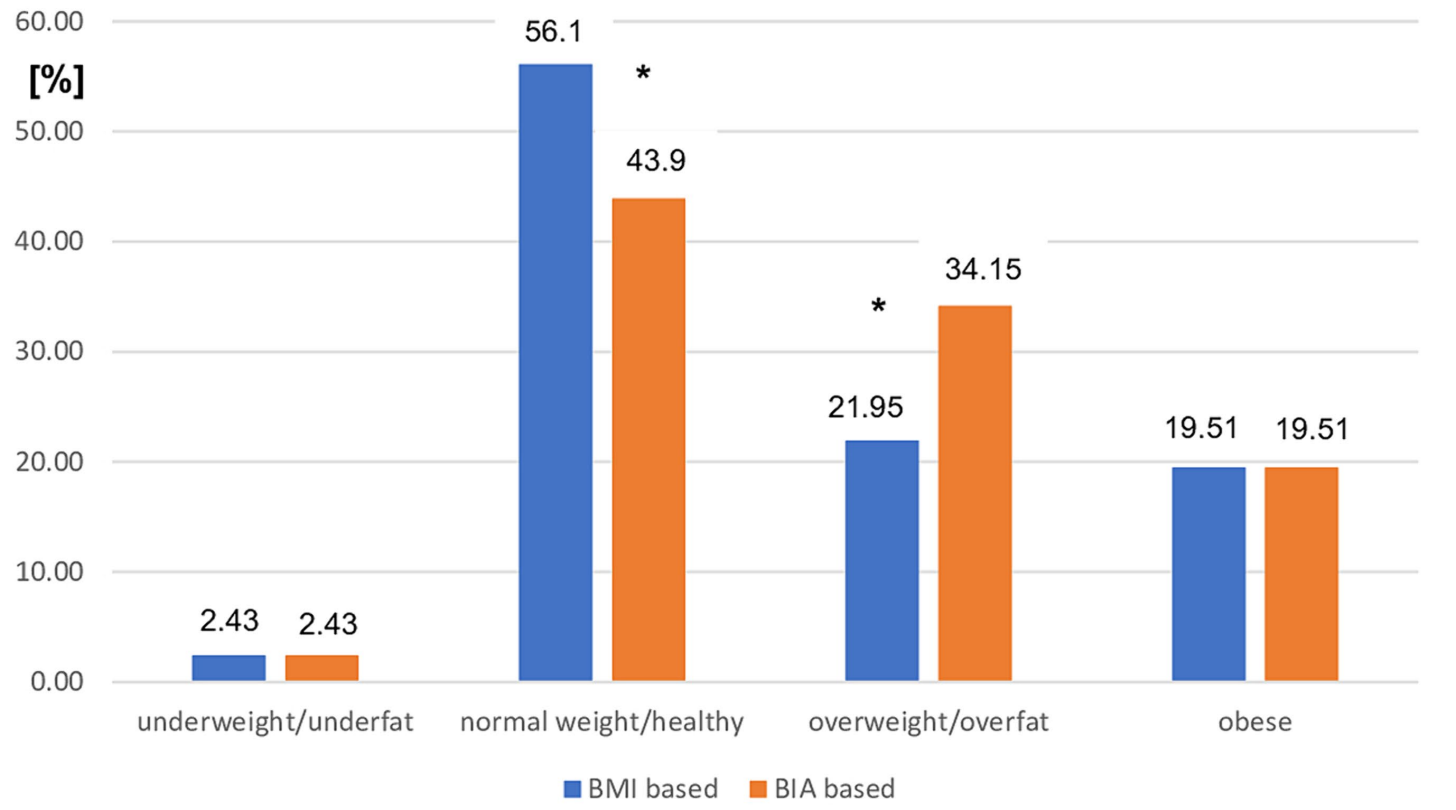
Prevalence of different nutritional status in moderate MS patients subgroup based on BMI vs. BIA. BMI, Body mass index; BIA, Bioelectrical impedance analysis; ^*^p < 0.01 (chi-square test).

However, the use of BMI cutoff points was related to a significant overestimation of normal weight status (*p* < 0.01) and underestimation of overweight status (*p* < 0.01) in patients with MS with moderate disability status assessed using EDSS (Figure 3).

### 3.4 Compatibility of nutritional status diagnoses based on BMI vs. BIA assessment in the mild and moderate disability subgroups of MS patients

The overall CR of diagnoses made using both BMI and BIA in patients with MS with mild disability was not significantly higher than that in the entire group (77.0 vs. 75.6%). The lowest CR was observed in the overweight/overfat group (Table 5). The lowest overall CR was observed in patients with moderately disabled MS. Only 70.7% of patients in this group had the same diagnoses (Tables 5, 6). Analyzing the two-sided CR between both modes of nutritional status assessment, significant differences were found for overweight/overfat diagnosis in both moderate and mild MS subgroups (*p* < 0.01 vs. *p* < 0.05, respectively) and for the normal weight/healthy category only in moderately disabled MS patients (*p* < 0.05) (Table 7).

**Table 5:** Interrelationship between different nutritional status diagnoses based on BMI vs. BIA in the mild MS subgroup.

Diagnosis by BMI (n)	Diagnosis by BIA (n)				*BMI all*
	Underfat	Healthy	Overfat	Obese	
Underweight	6	4	0	0	*10*
Normal	3	**65**	5	1	***74***
Overweight	0	3	**21**	5	***29***
Obesity	0	0	10	**12**	***22***
*BIA all*	***9***	***72***	***36***	***18***	**104/135 (77.0%)**

BMI, Body mass index; BAI, Bioimpedance analysis. Bold numbers refer to significant differences in the diagnosis of nutritional status between the methods used to assess it.

**Table 6:** Interrelationship between different nutritional status diagnoses based on BMI vs. BIA in the moderate MS subgroup.

Diagnosis by BMI (n)	Diagnosis by BIA (n)				*BMI all*
	Underfat	Healthy	Overfat	Obese	
Underweight	**1**	0	0	0	***1***
Normal	0	**16**	6	1	***23***
Overweight	0	2	**6**	1	***9***
Obesity	0	0	2	**6**	***8***
*BIA all*	***1***	***18***	***14***	***8***	**29/41 (70.7%)**

BMI, Body mass index; BAI, Bioimpedance analysis. Bold numbers indicate the concordance of diagnoses between the described methods used to identify adequate nutritional status.

**Table 7:** Two-sided compatibility rate (CR) in moderate and mild MS subgroups.

Nutritional status	Moderate MS (*n* = 41)	*p*	Mild MS (*n* = 135)		*p*	
	BMI vs. BIA	BIA vs. BMI		BMI vs. BIA	BIA vs. BMI	
Underweight/underfat	100% (1/1)	100% (1/1)	NS	60.0% (6/10)	66.7% (6/9)	NS
Normal/healthy	**69.6% (16/23)**	**88.9% (16/18)**	***p* < 0.05**	87.8% (65/74)	90.3% (65/72)	NS
Overweight/overfat	**66.7% (6/9)**	**42.9% (6/14)**	***p* < 0.01**	**72.4% (21/29)**	**58.3% (21/36)**	***p* < 0.05**
Obesity/obese	75% (6/8)	75% (6/8)	NS	54.5% (12/22)	66.7% (12/18)	NS

BMI, Body mass index; BAI, Bioimpedance analysis. *p* values were determined using the chi-square test. Bold numbers indicate the concordance of diagnoses between the described methods used to identify adequate nutritional status.

### 3.5 Nutritional status distribution measured by BMI vs. BIA after the stratification by abdominal obesity diagnosed as WHtR > 0.5

**Figure 4:**
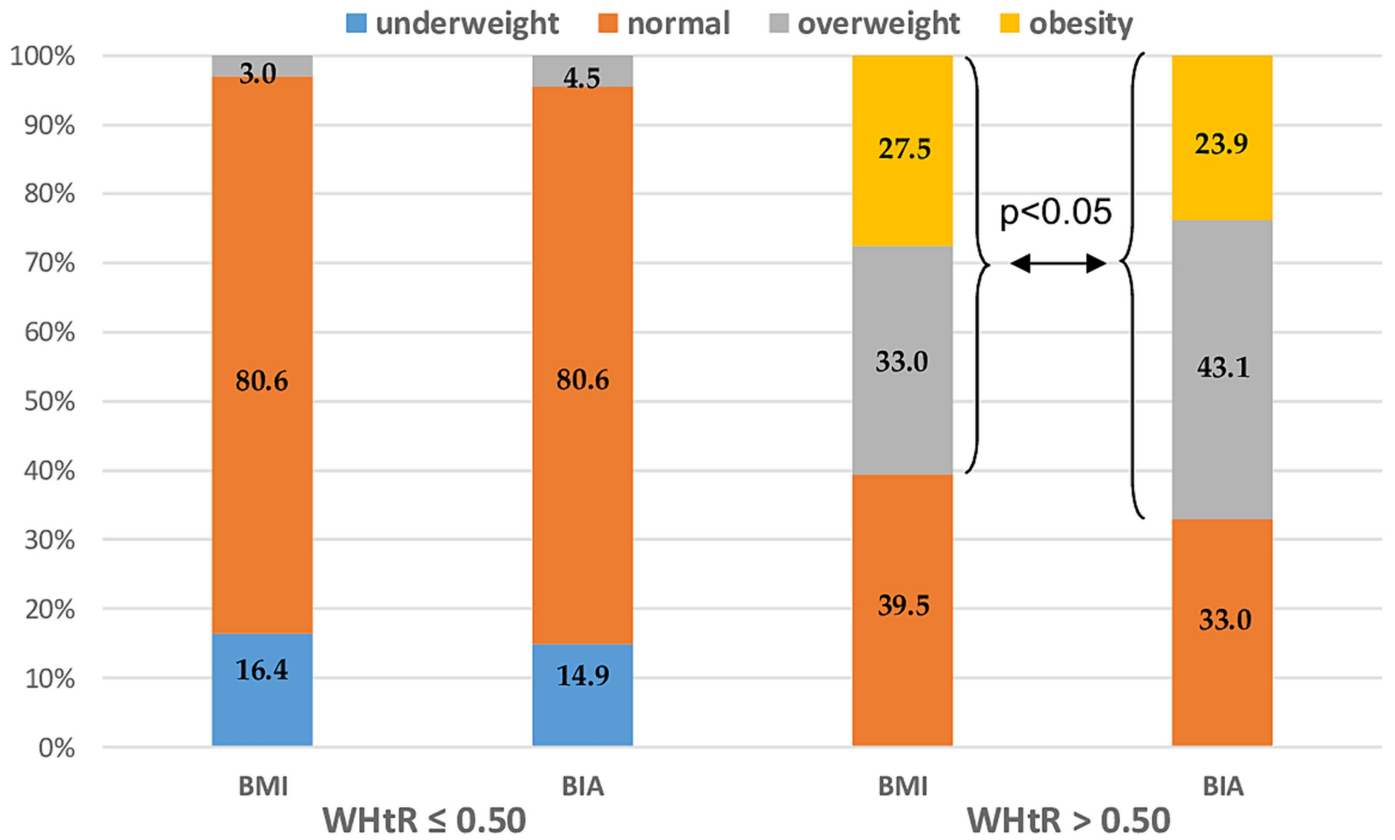
Nutritional status distribution measured by BMI vs. BIA after the stratification by abdominal obesity diagnosed as WHtR > 0.5. BMI, Body mass index; BIA, Bioelectrical impedance analysis; and WHtR, Waist-to-height ratio.

The study groups were stratified according to fat mass distribution. Abdominal obesity was diagnosed by calculating the waist-to-height ratio (WHtR). The cutoff point for the diagnosis of abdominal obesity was WHtR > 0.5. While, in the group of patients with WHtR ≤ 0.5 there were no significant differences between BMI vs. BIA assessment ones in the subgroup of patients with abdominal obesity (WHtR <0.5) BMI significantly underestimated the prevalence of MS patients with overweight and obesity vs. overfat and obese (60.5% vs. 67%; *p* < 0.05) (Figure 4).

### 3.6 Nutritional status distribution measured by BMI vs. BIA after the stratification by abdominal obesity

**Figure 5:**
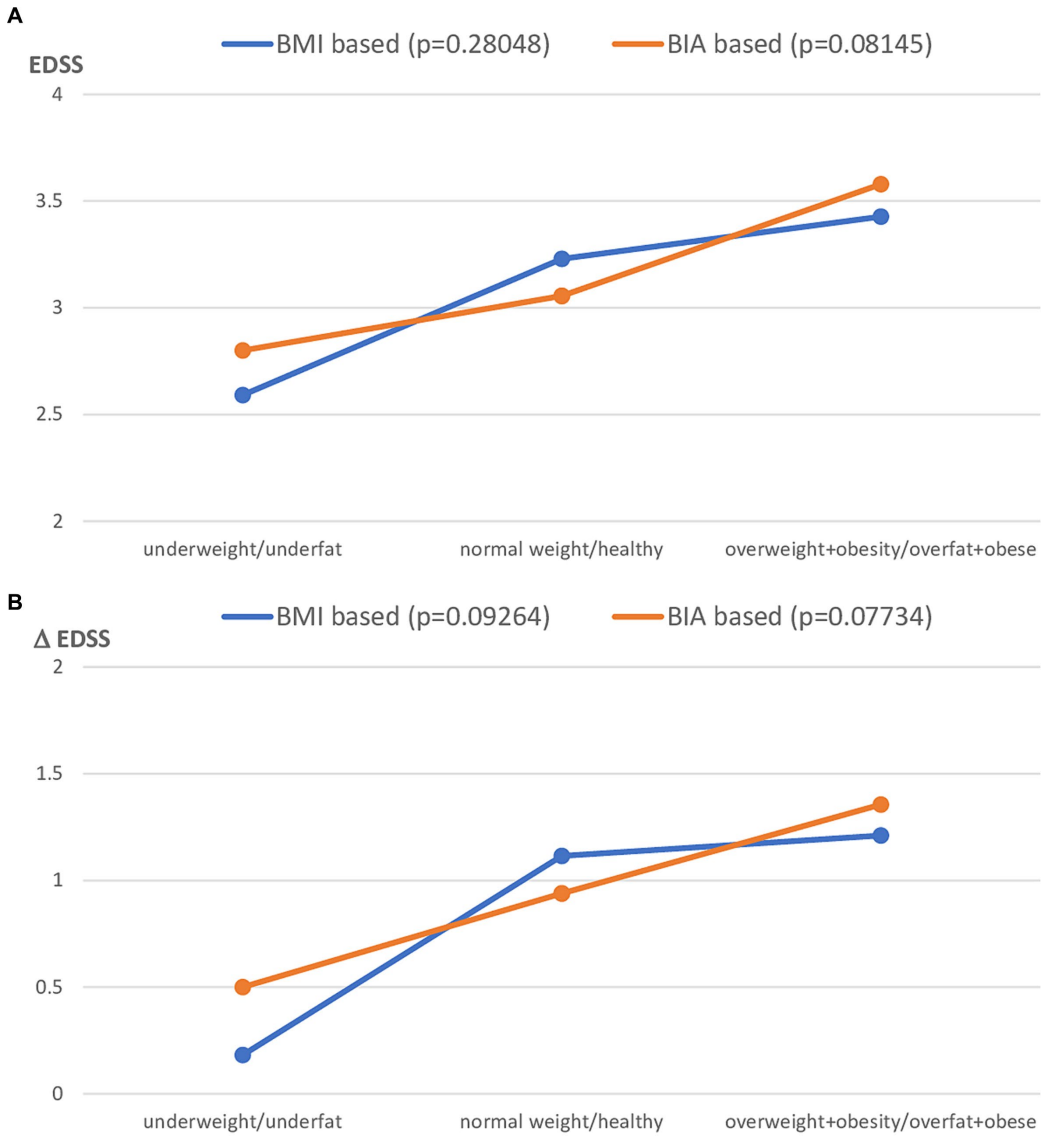
Clinical status of MS patients expressed as EDSS **(A)** and ΔEDSS **(B)**, after the stratification to the different nutritional status subgroups based on BMI vs. FAT% by BIA classification. BMI, Body mass index; BIA, Bioelectrical impedance analysis.

To analyze the interrelationship between both methods and the disability level of patients with MS, the present clinical status (assessed by EDSS) and MS progression (assessed by EDSS) were assessed. The study group was first stratified for the different nutritional status category based on both BMI and BIA and then one-way ANOVA was performed for EDSS and ΔEDSS, respectively. Both EDSS and ΔEDSS were more related to the nutritional status categorized by FAT% assessed by BIA than using BMI cut-off points. However, this relationship was not statistically significant (Figure 5A for EDSS and Figure 5B for ΔEDSS).

## 4 Discussion

In our study, we assessed the consistency of the distribution of nutritional status categories based on BMI cutoff points and bioelectrical impedance analysis (BIA) by fat mass percentage (FM%). We observed that FM assessed by BIA revealed a significantly lower prevalence of underfat and healthy persons; however, there was a significantly higher prevalence of overfat patients with MS than overweight patients based on the BMI cutoff point. However, the prevalence of obesity did not differ significantly, regardless of the method of diagnosis, and was not significantly lower when assessed using BIA. The overall compatibility rate (CR) of the diagnoses made using both BMI and BIA was 75.6% in the entire patient group. After stratification for disability status (based on the EDSS), a similar CR was found for patients with MS in the mild disability group (77.0%). However, the CR was distinctly lower in patients in the moderate MS subgroup. The distribution of nutritional status categories diagnosed by both BMI and BIA cutoff points in patients with MS with mild disability was similar to that in the entire study group. Again, there was a significant difference between the prevalence of overweight (assessed by BMI) and the overfat category assessed by BIA. However, the use of BMI cut-off points was related to a significant overestimation of normal weight status and underestimation of overweight status in MS patients with moderate disability. Analyzing the two-sided CR between both methods of nutritional status assessment, significant differences were found for overweight/overfat diagnosis in both moderate and mild MS subgroups, and for the normal weight/healthy category only in moderately disabled MS patients.

Our findings confirm the data from the study by Hedström et al. (4), which showed that BMI assessment may cause an underestimation of adiposity in patients with MS compared with body composition measured by DXA. The same conclusions were drawn by Wingo et al. (15), who showed significantly higher fat mass and lower fat-free mass in men with MS than in healthy BMI-matched controls. The limitation of using BMI in patients with MS was also presented in a study showing a relationship between MS and longitudinal changes in BMI (16). The authors make three main observations. Baseline BMI in patients with MS was significantly higher than that in healthy controls; BMI was significantly higher in healthy controls with increasing age, and there were no longitudinal associations between BMI and EDSS. A recently published study postulated the use of a simple model to estimate the percentage of body fat in individuals with MS based only on BMI and sex using a special mathematical formula. However, it was cross-validated with DXA body composition in only 33 patients with MS (six males) and was not related to disability status (17). Dual-energy X-ray absorptiometry (DXA) is currently the gold standard for diagnosing osteoporosis and evaluating fat mass. However, a noninvasive body composition assessment technique based on bioelectrical impedance analysis (BIA) is currently available. Good correlation between BIA and DXA has been reported for estimating both fat mass and fat-free mass in different populations (18, 19). BIA is a relatively inexpensive, quick, simple, readily accessible, and non-invasive technique. Body composition analysis using BIA in patients with MS has been used in two studies that focused mainly on nutritional intake rather than on nutritional status (20, 21). The MS patient group was relatively small (*n* = 20 and *n* = 37, respectively) MS patients and the body fat percentage assessed by BIA was only shown as a mean result for the studied population; however, the authors did not show any detailed results related to body composition.

In analyzing fat tissue, it is important to remember that, from a metabolic point of view, body fat distribution has great importance and is closely related to the special risk for abdominal/visceral obesity, which cannot be properly assessed by BMI itself. The waist-to-height ratio (WHtR) is now widely studied to determine relatively simple parameters of fat tissue distribution in connection with visceral obesity and its comorbidities. A recent analysis showed that WHtR is the best parameter for the prognosis of visceral fat and its comorbidities (22). Our recently published study showed a significant correlation between this parameter and the disability status, disease duration, and glucocorticoid use in patients (12). Moreover, in a study performed by Cozart et al. (23), a higher waist-to-height ratio (WtHR) was associated with worse physical performance outcomes in patients with MS, as measured by the 6-min walk test (6 MWT) and the Timed 25 Foot Walk (T25FW). Another aspect of the potential influence of adiposity on MS progression is the generation of adipose tissue-related inflammation and oxidative stress. A recent study by Drehmer et al. (24) revealed a significant correlation between fat mass distribution assessed by both waist circumference and WHtR and oxidative stress and inflammation markers in obese patients with MS. In the present study, the study group was stratified according to the diagnosis of abdominal obesity, which was assessed by calculating the calculation of waist-to-height ratio (WHtR). Leg-to-leg BIA methodology does not provide information on fat mass distribution. In the group of patients with a normal WHtR, there were no significant differences between BMI and BIA assessment in the subgroup of patients with abdominal obesity (WHtR < 0.5), and BMI significantly underestimated the prevalence of overweight and obesity vs. overweight and obese patients.

In the present study, we found that clinical status and disease progression assessed by EDSS were more closely related to nutritional status diagnosed by body composition (BIA) than to standard anthropometrical diagnosis based on BMI. However, the relationship was not statistically significant. These findings are similar to those reported by Pilutti et al. (25), who revealed that FM assessed using DXA was significantly higher in MS patients with moderate disability. Moreover, they did not find a significant difference in BMI between the mild and moderate disability groups. The lack of a significant correlation between BMI and disability status scores has recently been confirmed by several authors (12, 16, 25). Our recently published studies also showed a significant correlation between body composition parameters (FM% and FFM%) and disability status in patients (11, 12). However, conflicting results regarding the lack of significant correlations between the EDSS and BMI, Waist c., WHR, and FM% from BIA were noted in a group of 137 Brazilian patients (26).

Body composition parameter assessment seems to be important because in patients with MS, the risk of sarcopenia related to the level of disability is very high. A study conducted by Wens et al. (27) revealed a higher fat percentage and lower lean mass in muscle biopsies of patients with MS. Other data published by Ward et al. (28) and Wingo et al. (15) showed that higher FM% and lower FFM% were associated with lower limb physical function, suggesting that body composition, specifically reducing adiposity and increasing lean mass, may be a potential target for MS interventions. It is also important to realize that lean mass is strongly related to the bone mineral density (BMD), either in whole body and lumbar spine projection (29).

This study has some limitations. First, the number of patients was relatively small, especially in the moderate MS subgroup. Second, data describing the initial nutritional status of patients with MS are lacking. These limitations indicate that future studies should focus on prospective longitudinal body composition assessments in larger cohorts of MS patients.

## 5 Conclusion

Using BMI cutoff points for assessing the nutritional status in patients with MS is associated with a significant underestimation of excess fat mass. BIA-based FAT% based on BIA have a better relationship with abdominal obesity and disability status than with BMI in patients with MS. The highest rate of false-negative diagnoses was based on the BMI in patients with MS and moderate disability. Adiposity assessment using BIA appears to be a useful method for proper nutritional status assessment in the patients group.

## Data availability statement

The raw data supporting the conclusions of this article will be made available by the authors, without undue reservation.

## Ethics statement

The studies involving humans were approved by Ethics Committee of the Medical University of Silesia (Approval No. KNW/0022/KB/179/17). The studies were conducted in accordance with the local legislation and institutional requirements. The participants provided their written informed consent to participate in this study.

## Author contributions

EM: Writing – review & editing, Writing – original draft, Validation, Supervision, Resources, Project administration, Methodology, Investigation, Funding acquisition, Formal analysis, Data curation, Conceptualization.
